# Supplementary material for: Prognostic Significance of Interim Response Evaluation during Definitive Chemoradiotherapy for Locally Advanced Esophageal Squamous Cell Carcinoma
Source: Cancers (Basel). 2021 Mar 12;13(6):1255. doi: 10.3390/cancers13061255 (PMC8000322; doi:10.3390/cancers13061255)
Supplement: Supplementary file 1 [file cancers-13-01255-s001.pdf]

cancers

MDPI

Supplementary Materials

Prognostic Significance of Interim Response Evaluation during Definitive Chemoradiotherapy for Locally Advanced Esophageal Squamous Cell Carcinoma

Jun Gi Yeom, Jie-Hyun Kim, Jun Won Kim, Yeona Cho, Ik Jae Lee, Chang Geol Lee, Jaeyoung Chun, Young Hoon Youn and Hyojin Park

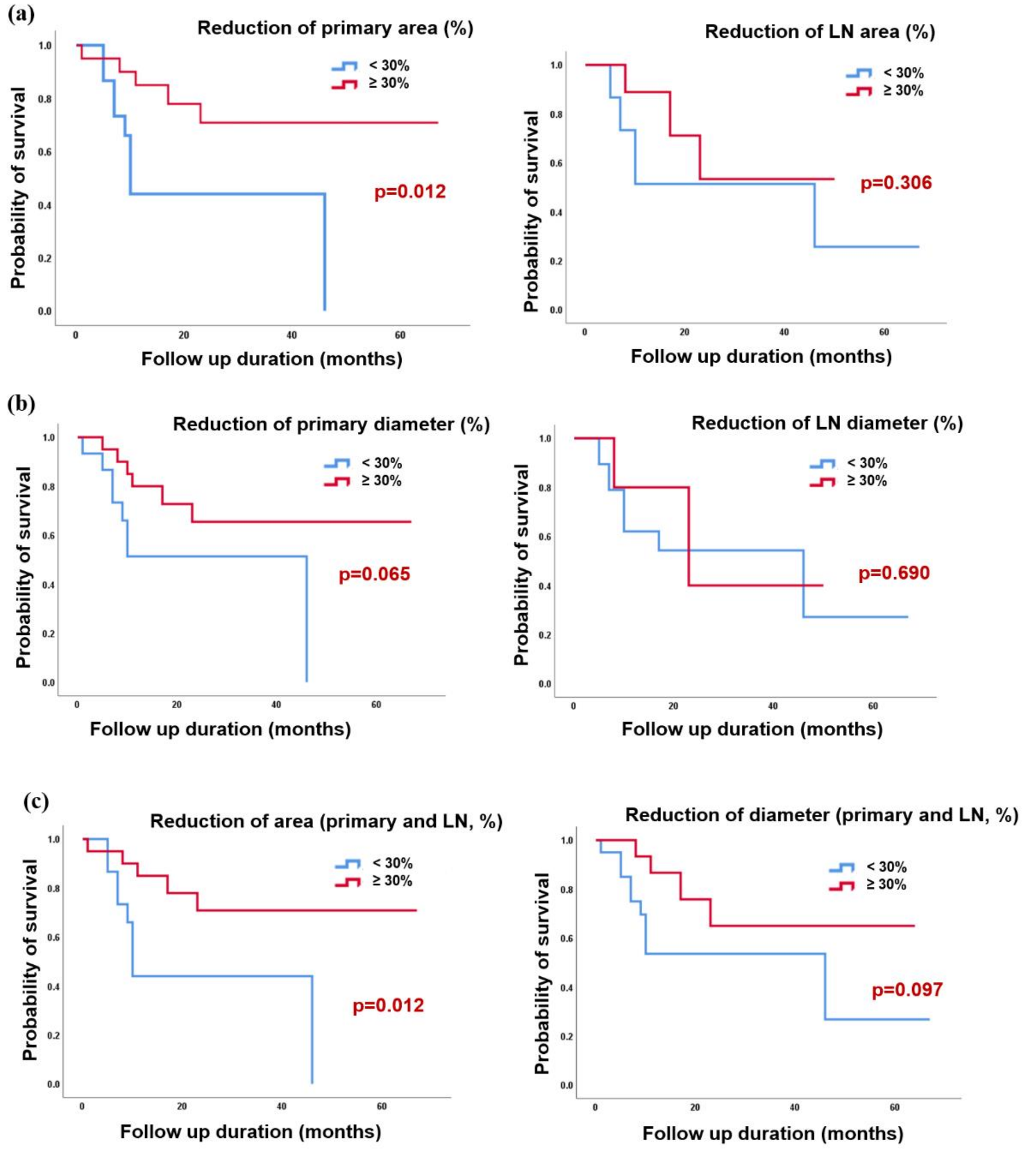

Figure S1. Kaplan–Meier analysis of progression-free survival (PFS) rates shows a relationship between the reduction of lesion size and PFS. (**a**) Area reduction of the primary esophageal le-sion/lymph node (LN) and PFS rates; (**b**) Diameter reduction of the primary esophageal lesion/LN and PFS rates; (**c**) Area and diameter reduction of both lesions (sum of pri-mary esophageal lesion and LN) and PFS rates.
